# A systems-level energetic framework for conscious processing: energetic organization as a constraint on large-scale neural integration

**DOI:** 10.3389/fnhum.2026.1966072

**Published:** 2026-10-23

**Authors:** Holly Pollard-Wright

**Affiliations:** Independent Researcher, Rialto, CA, United States

**Keywords:** brain energy metabolism, consciousness, consciousness theories, functional connectivity, large-scale neural integration, network neuroscience, neuroenergetics, systems neuroscience

## Abstract

Conscious processing is associated with coordinated interactions among distributed neural systems, yet the biological constraints that enable and sustain large-scale neural integration remain incompletely understood. Contemporary theories emphasize computational, informational, and network mechanisms, while the potential contribution of systems-level energetic organization has received comparatively less explicit theoretical attention. This Hypothesis & Theory article proposes that systems-level energetic organization may represent an additional biological constraint influencing the neural conditions under which large-scale neural integration associated with conscious processing can emerge and be sustained. Systems-level energetic organization is defined as the dynamic coordination of energy production, distribution, allocation, and utilization across interacting neural systems. The framework integrates established principles of brain energetics, metabolic regulation, and distributed neural organization, while the proposed relationship between their systems-level coordination and conscious neural integration remains hypothetical. It is intended to complement rather than replace existing theories of consciousness and does not propose that energy generates consciousness, determines conscious content, or explains subjective experience. The framework generates experimentally testable predictions concerning whether systems-level energetic measures provide explanatory value beyond regional metabolism and established neurophysiological and network variables. Explicit falsification criteria define observations that would weaken or reject the hypothesis. Multimodal human and non-human animal studies, computational modeling, and carefully controlled translational investigations provide complementary approaches for evaluating these predictions. By treating systems-level energetic organization as an empirically testable biological constraint rather than an explanation for consciousness itself, the proposed framework provides a falsifiable research program for investigating an unresolved dimension of large-scale neural integration while remaining agnostic regarding the ultimate origin and nature of subjective experience.

## 1 Introduction

Consciousness remains one of the most challenging and unresolved problems in neuroscience. Over the past several decades, substantial progress has been made in identifying the neural activity associated with conscious processing, and numerous theoretical frameworks have advanced understanding of information integration, large-scale communication, predictive computation, recurrent processing, and network organization (Bullmore and Sporns, 2009; Friston, 2010; Mashour et al., 2020; Seth and Bayne, 2022). Despite these advances, no consensus framework fully explains how distributed neural activity becomes organized into the large-scale neural integration associated with conscious processing (Mashour et al., 2020; Modolo et al., 2020). At the same time, comparatively little attention has been devoted to whether systems-level energetic organization itself constrains the large-scale neural integration required for conscious processing. This remaining uncertainty motivates investigation of whether a biologically grounded systems-level energetic framework offers a complementary, experimentally testable perspective on this unresolved question.

A central principle of neuroscience is that all neural activity is constrained by energy. Although the brain represents only a small fraction of total body mass, it consumes a disproportionate amount of the body’s metabolic energy to sustain neuronal signaling, synaptic transmission, and large-scale network communication. Consequently, all neural computation operates within fundamental energetic constraints (Attwell and Laughlin, 2001; Raichle and Gusnard, 2002; Harris et al., 2012; Rae et al., 2024). Although these energetic principles are well established, how systems-level energetic organization may constrain the organization of large-scale neural integration remains considerably less well understood.

This distinction defines the central scientific gap addressed in this manuscript. Contemporary theories provide important explanations for how information is represented, integrated, and communicated across distributed neural networks (Tononi et al., 2016; Mashour et al., 2020; Modolo et al., 2020). However, considerably less attention has been devoted to whether the energetic organization required to sustain these distributed interactions represents a systems-level constraint on conscious processing. The absence of such a framework does not imply that existing theories are incomplete or incorrect. Rather, it identifies a complementary scientific question that has yet to be developed into a biologically plausible and experimentally testable conceptual framework.

The objective of this Hypothesis & Theory article is to propose a biologically plausible and experimentally testable systems-level energetic framework that addresses this unresolved question. Specifically, the author hypothesizes that conscious experience is constrained by systems-level energetic organization, suggesting that a coherent energetic framework may help explain how distributed neural activity supports the large-scale integration required for conscious processing while remaining agnostic regarding the ultimate origin or nature of subjective experience (Modolo et al., 2020). Importantly, this hypothesis does not propose that energy generates consciousness, nor does it claim to solve the “hard problem” of consciousness. Instead, the proposed framework suggests that systems-level energetic organization may represent an additional biological constraint on the neural conditions required for conscious processing. This perspective generates experimentally testable predictions intended to complement, rather than replace, existing theories of consciousness.

The manuscript begins by reviewing established evidence concerning brain energetics, large-scale neural integration, functional connectivity, oscillatory dynamics, and metabolic constraints relevant to conscious processing. It then introduces the proposed systems-level energetic framework, examines its relationship to existing theories of consciousness, develops experimentally testable predictions, defines falsification criteria, and outlines future experimental directions. Throughout, the manuscript distinguishes established evidence from interpretation, hypothesis, and speculation while maintaining scientific restraint expected of a *Hypothesis & Theory* article. Its objective is to provide a biologically plausible, experimentally testable framework that can be evaluated, refined, or rejected through future empirical investigation.

## 2 Established scientific background

The proposed hypothesis is founded on established principles of neuroscience rather than the introduction of new biological mechanisms. Understanding whether systems-level energetic organization contributes to conscious processing first requires consideration of the established evidence governing brain energetics, large-scale neural integration, functional connectivity, oscillatory dynamics, and metabolic constraints. This section therefore summarizes the current scientific foundation upon which the proposed framework is built while carefully distinguishing established evidence from subsequent interpretation and hypothesis (Seth and Bayne, 2022).

A fundamental principle of neuroscience is that all neural activity is constrained by energy. Although the brain represents only a small fraction of total body mass, it consumes a disproportionately large share of the body’s metabolic energy to sustain neuronal signaling, synaptic transmission, intracellular signaling, neurotransmitter recycling, maintenance of ionic gradients, and cellular homeostasis. Consequently, all neural computation operates within fundamental energetic constraints. These energetic demands are inherent properties of normal brain physiology and establish the biological context within which all higher-order neural processing occurs (Attwell and Laughlin, 2001; Raichle and Gusnard, 2002; Harris et al., 2012; Rae et al., 2024).

Brain energetics are dynamically regulated to match continuously changing computational demands. Regional increases in neuronal activity are accompanied by tightly coordinated adjustments involving neurons, astrocytes, cerebral blood flow, glucose utilization, and mitochondrial ATP production. Through neurovascular and neurometabolic coupling, energy delivery is continuously matched to local functional requirements, allowing distributed neural networks to maintain normal physiological activity. These well-established processes demonstrate that energetic regulation is an active, highly coordinated component of normal brain function rather than a passive background condition (Attwell et al., 2010; Harris et al., 2012; Rae et al., 2024).

Conscious processing is likewise associated with coordinated interactions among distributed neural populations rather than isolated regional activity. Evidence from functional neuroimaging, electrophysiology, and network neuroscience demonstrates that conscious states are accompanied by dynamic communication across widespread cortical and subcortical networks (Koch et al., 2016; Mashour et al., 2020; Modolo et al., 2020). Conversely, transitions associated with general anesthesia, disorders of consciousness, non-rapid eye movement sleep, and other altered conscious states are frequently accompanied by reductions in large-scale functional connectivity, impaired network integration, or disrupted long-range communication. Although the precise mechanisms underlying these observations remain under active investigation, they consistently demonstrate a strong association between conscious processing and coordinated interactions extending across distributed brain networks (Koch et al., 2016; Mashour et al., 2020).

Large-scale neural integration depends upon multiple complementary organizational principles. Structural connectivity provides the anatomical architecture through which distributed communication occurs, functional connectivity reflects the dynamic interactions among neural populations, and oscillatory synchronization facilitates the temporal coordination of activity across multiple spatial and temporal scales. Together, these complementary properties enable efficient communication throughout the brain while remaining subject to the biological constraints imposed by underlying cellular physiology (Bullmore and Sporns, 2009; Friston, 2010; Rubinov and Sporns, 2010).

An additional established principle of systems neuroscience is that brain networks are organized to balance efficient communication with biological cost. Network architecture reflects trade-offs among wiring distance, communication efficiency, metabolic demand, and functional specialization rather than maximizing any single property independently. Consequently, efficient large-scale neural communication is achieved within anatomical, energetic, and physiological constraints. These observations further demonstrate that biological constraints influence the organization of distributed brain networks at multiple levels of neural organization (Bassett and Bullmore, 2006; Bullmore and Sporns, 2009; Rubinov and Sporns, 2010).

Collectively, these established findings demonstrate that neural computation, large-scale communication, and distributed network integration all require continuous energetic support. However, current evidence primarily establishes that adequate energy availability is necessary for normal brain function. It does not establish whether the systems-level organization of energy utilization itself contributes directly to the large-scale neural integration required for conscious processing. Consequently, although contemporary neuroscience provides a robust understanding of brain energetics, network organization, and large-scale communication, the potential relationship between systems-level energetic organization and conscious neural integration remains incompletely understood (Bullmore and Sporns, 2009; Modolo et al., 2020; Rae et al., 2024).

The proposed relationship is not assumed to be unique to consciousness. Energetic constraints apply broadly to neural computation, including processes that occur without conscious awareness. The present framework focuses specifically on conscious processing as a defined domain in which changes in large-scale neural integration can be examined across distinguishable conscious states and conditions. Accordingly, the hypothesis is not that systems-level energetic organization is exclusive to consciousness or distinguishes conscious from all nonconscious neural processing, but that it may constrain the capacity for the distributed neural integration associated with conscious processing. Whether comparable energetic organizational relationships also constrain other forms of large-scale neural computation, and whether any features demonstrate specificity for conscious processing, remain empirical questions.

Existing experimental studies provide partial empirical precedent for a relationship between energetic state and coordinated neural activity, although they do not directly test the present systems-level hypothesis. ATP dynamics have been measured in electrically active neural tissue and shown to vary in relation to neural activity, providing direct evidence of dynamic coupling between cellular energetic state and electrical function (Trevisiol et al., 2017). In a complementary perturbational study, mild metabolic stress disrupted the formation of coordinated pyramidal-cell ensembles during gamma oscillations, demonstrating that altered energetic conditions can affect organized neural population activity (Elzoheiry et al., 2020). These findings provide empirical support for coupling between energetic state and coordinated neural dynamics but do not establish that spatially and temporally coordinated energetic organization across distributed neural systems independently constrains large-scale neural integration or conscious processing. Direct tests of that relationship remain lacking and constitute the empirical gap addressed by the present framework.

Collectively, these established findings provide the scientific foundation for considering whether systems-level energetic organization represents more than a permissive requirement for neural activity. The following section introduces the proposed systems-level energetic framework, carefully distinguishing established evidence from conceptual interpretation while explicitly identifying the aspects of the framework that remain hypothetical.

## 3 Proposed energetic framework

The preceding sections established that brain function depends upon continuous energetic support and that conscious processing is consistently associated with coordinated interactions among distributed neural networks. They also identified an unresolved question: whether systems-level energetic organization contributes more directly to the large-scale neural integration required for conscious processing. The framework proposed here addresses this unresolved question by introducing a systems-level energetic perspective on large-scale neural integration. It does not claim that energy generates consciousness, nor does it propose a complete theory of consciousness. Rather, it advances the hypothesis that the coordinated organization of energetic resources across distributed neural systems constrains the capacity for large-scale neural integration associated with conscious processing (Mashour et al., 2020).

For the purposes of this manuscript, systems-level energetic organization refers to the dynamic coordination of energy production, distribution, allocation, and utilization across interacting neural systems. The proposed framework distinguishes systems-level energetic organization from local cellular metabolism by emphasizing the coordinated organization of energetic resources across distributed neural systems rather than energy utilization within individual neurons or individual brain regions. It therefore focuses on how energetic resources may be organized across distributed networks in ways that support the coordinated neural interactions consistently associated with conscious processing (Harris et al., 2012; Dienel, 2019; Mashour et al., 2020).

Within this framework, systems-level energetic organization refers to the spatial and temporal patterning and coordination of energetic states across distributed neural populations, rather than simply to total energy availability, ATP abundance, mitochondrial abundance, or metabolic activity within individual regions. The proposed relationship therefore concerns how established energetic processes are coordinated across distributed neural systems in relation to the physiological demands of large-scale neural integration, rather than the transfer of energetic resources between distant regions. The hypothesis is that this systems-level organization may constrain the capacity for distributed neural populations to sustain coordinated interactions; whether it provides explanatory information beyond the neural activity it supports remains an empirical question. This proposed relationship is not restricted to pathological energetic failure but may be evaluated across normal physiological variation and experimentally or clinically altered states.

Energetic constraints are defined as the physiological limitations imposed by the finite availability, distribution, and regulation of metabolic resources required to sustain neural activity. These constraints arise from established biological processes, including mitochondrial ATP production, neurovascular coupling, substrate delivery, and cellular metabolic regulation. The proposed hypothesis does not suggest that these mechanisms are novel. Rather, it proposes that their coordinated organization across distributed neural systems may influence the capacity of the brain to maintain the large-scale neural integration associated with conscious processing (Attwell and Laughlin, 2001; Attwell et al., 2010; Rae et al., 2024).

Conceptually, the proposed framework views energetic organization as a systems-level constraint within which neural computation occurs. Neural signaling, information processing, oscillatory synchronization, and large-scale network communication all require continuous metabolic support (Laughlin and Sejnowski, 2003; Harris et al., 2012). Consequently, although existing theories emphasize information flow, network dynamics, predictive computation, recurrent processing, or integrated information, these processes necessarily operate within biological limits imposed by energetic availability, allocation, and utilization. The proposed framework therefore suggests that energetic organization should be considered as an additional systems-level constraint operating alongside these established mechanisms rather than as an alternative explanation.

The novelty of the proposed framework lies not in introducing new mechanisms of energy metabolism or neural communication, both of which are well established, but in hypothesizing that their coordinated organization across distributed neural systems represents an experimentally testable systems-level constraint on the neural integration associated with conscious processing (Mashour et al., 2020).

The proposed framework is summarized in Figure 1.

**Figure 1:**
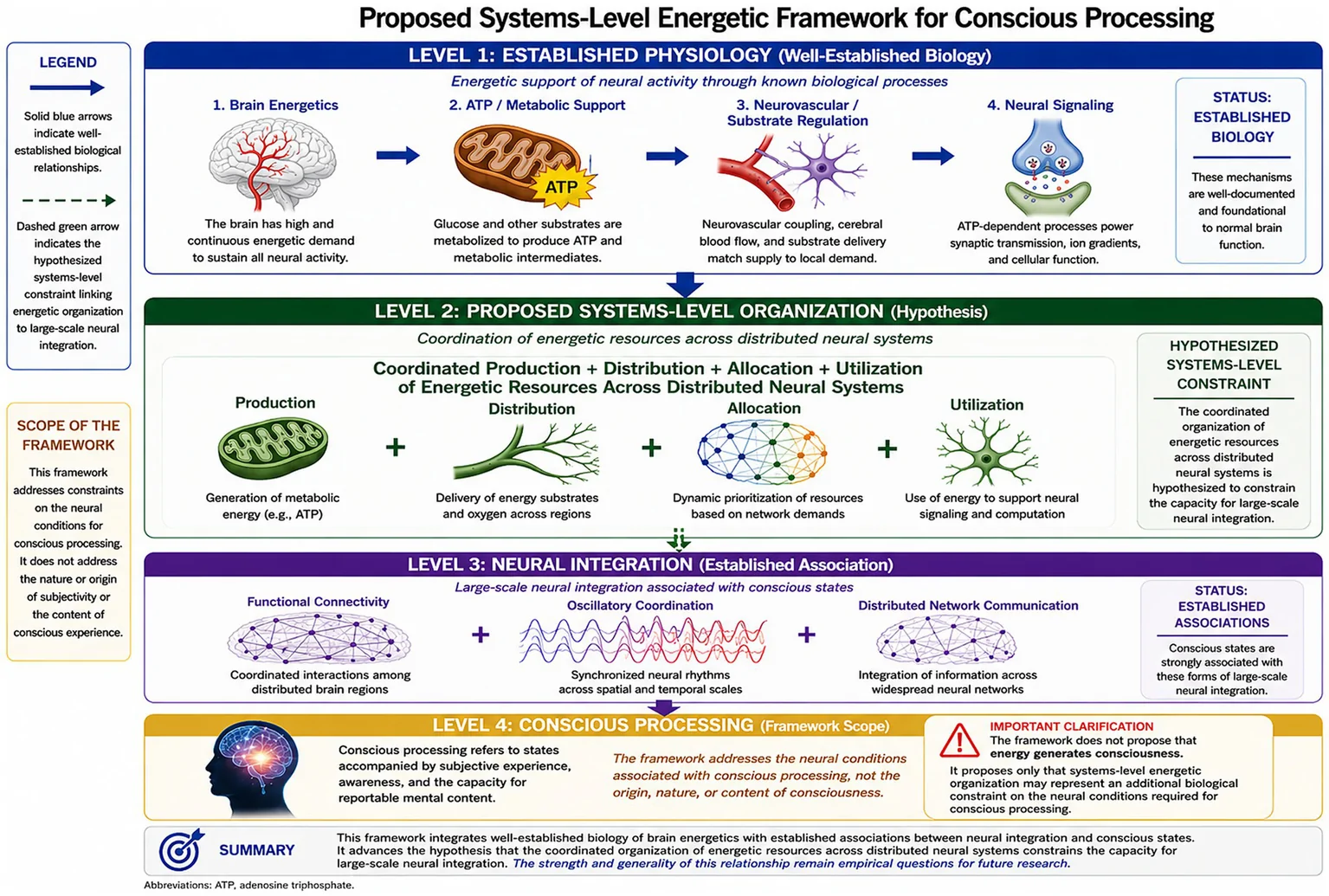
Proposed systems-level energetic framework for conscious processing. Schematic representation of the proposed relationship between established brain energetics, systems-level energetic organization, large-scale neural integration, and conscious processing. Level 1 summarizes established physiological processes supporting neural activity, including brain energetic demand, ATP and metabolic support, neurovascular and substrate regulation, and energy-dependent neural signaling. Level 2 represents the central hypothesis of the framework: The coordinated production, distribution, allocation, and utilization of energetic resources across distributed neural systems is hypothesized to constrain the capacity for large-scale neural integration. The dashed green arrow denotes this hypothesized systems-level constraint and distinguishes it from established physiological relationships. Level 3 depicts established associations between conscious states and large-scale neural integration, including functional connectivity, oscillatory coordination, and distributed network communication. Level 4 defines conscious processing as the scope of the framework. The framework addresses biological constraints on the neural conditions associated with conscious processing; it does not propose that energy generates consciousness or explain the origin, nature, or content of subjective experience. ATP, adenosine triphosphate.

Importantly, this hypothesis does not imply that energetic organization determines the content of conscious experience or explains the subjective nature of consciousness. Nor does it imply that energy alone is sufficient to generate consciousness. The framework proposes only that the coordinated organization of energetic resources across distributed neural systems is hypothesized to constrain the capacity for large-scale neural integration associated with conscious processing (Attwell et al., 2010). Whether this relationship exists, and the extent to which it contributes to conscious neural function, remains an empirical question requiring experimental investigation.

This framework is biologically plausible because it is constructed entirely from established physiological principles rather than the introduction of new biological mechanisms. Brain energetics, neurovascular regulation, mitochondrial function, and large-scale neural communication are all well-established components of contemporary neuroscience. Rather than introducing new biological mechanisms, the proposed framework integrates these established physiological principles into a systems-level conceptual model that generates experimentally testable questions regarding their coordinated organization. In this respect, the framework represents an interpretive synthesis of existing knowledge rather than the proposal of previously unknown biological processes (Bullmore and Sporns, 2009; Attwell et al., 2010; Dienel, 2019; Rae et al., 2024).

Several important uncertainties should be acknowledged explicitly. At present, there is no direct evidence demonstrating that systems-level energetic organization independently constrains the large-scale neural integration associated with conscious processing. Likewise, the proposed framework does not specify the precise mechanisms by which energetic organization might influence large-scale neural integration, nor does it exclude alternative explanations provided by existing theories of consciousness. These limitations are intentional and consistent with the objectives of a Hypothesis & Theory article. The value of the present framework therefore lies not in claiming explanatory completeness, but in proposing a biologically plausible hypothesis that can be evaluated through empirical investigation.

## 4 Relationship to existing theories

The proposed systems-level energetic framework is intended to complement, rather than replace, existing theories of consciousness. Contemporary models have substantially advanced understanding of the neural mechanisms associated with conscious processing by emphasizing information integration, global communication, predictive computation, recurrent interactions, and large-scale network dynamics. These contributions remain fundamental to contemporary neuroscience. Rather than competing with these theories, the proposed framework asks whether systems-level energetic organization represents an additional biological constraint operating alongside the mechanisms they describe. In doing so, it seeks to extend rather than redefine current theoretical perspectives (Seth and Bayne, 2022).

Among the most influential contemporary models, Global Workspace Theory (GWT) proposes that conscious processing arises when information becomes globally available through widespread communication among distributed neural systems (Baars, 1988; Dehaene and Naccache, 2001; Mashour et al., 2020). The proposed energetic framework is fully consistent with the central premise that large-scale neural communication is essential for conscious processing. However, whereas GWT primarily addresses how information is broadcast across distributed networks, the present framework considers whether the coordinated energetic organization required to sustain that widespread communication may itself constrain the biological conditions under which large-scale broadcasting can occur. Energetic organization therefore complements, rather than replaces, the mechanisms proposed by GWT.

Similarly, Integrated Information Theory (IIT) proposes that consciousness is related to the intrinsic cause–effect structure of a physical system and its capacity for integrated information (Tononi et al., 2016). The energetic framework neither supports nor challenges IIT’s mathematical formulation of integrated information. Instead, it proposes that achieving and maintaining highly integrated neural states necessarily occurs within physiological energetic constraints. Accordingly, the present framework focuses on the physiological organization required to sustain distributed neural integration rather than on quantifying integrated information itself.

Predictive Processing conceptualizes the brain as a hierarchical inference system that continuously minimizes prediction error through reciprocal interactions among distributed neural networks (Rao and Ballard, 1999; Clark, 2013). This framework has generated substantial insight into perception, cognition, and adaptive behavior. The proposed energetic framework is compatible with these concepts while suggesting that predictive computations necessarily operate within coordinated systems-level energetic constraints. Under this interpretation, energetic organization neither replaces predictive processing nor alters its computational principles but may contribute to defining the physiological conditions under which predictive neural processing is sustained.

Likewise, Recurrent Processing Theory (RPT) emphasizes the importance of recurrent cortical interactions in generating conscious perception (Lamme, 2006). The energetic framework remains fully compatible with this perspective because recurrent communication requires sustained neural activity across distributed neural circuits. Rather than offering an alternative explanation, the present hypothesis proposes that coordinated energetic organization may contribute to maintaining the physiological conditions necessary for recurrent neural interactions to occur over time.

Higher-Order theories propose that conscious awareness depends on higher-order representations of, or relations to, first-order mental or neural states (Lau and Rosenthal, 2011). The present energetic framework does not address the representational requirements proposed by these theories, but asks whether the distributed neural interactions supporting higher-order processing operate within measurable systems-level energetic constraints. Related active-inference approaches characterize conscious processing in terms of hierarchical generative models, inference, and the regulation of interactions between the organism and its environment (Laukkonen et al., 2025). The present framework does not replace these computational principles; rather, it asks whether their neural implementation operates within coordinated physiological energetic constraints across distributed neural systems.

Although these theories differ substantially in their explanatory commitments, each describes organizational or computational properties of neural systems that have been proposed to contribute to conscious processing. Across the theories considered here, these properties include global availability, information integration, predictive computation, and recurrent neural interactions (Seth and Bayne, 2022). None, however, explicitly places systems-level energetic organization at the center of its conceptual framework as an experimentally testable systems-level constraint on distributed neural integration. The novelty of the proposed framework therefore lies not in redefining existing theories of consciousness, but in proposing that systems-level energetic organization itself constitutes an experimentally testable physiological dimension that may operate across multiple theoretical models.

Scientific progress frequently results from integrating complementary explanatory perspectives rather than replacing established frameworks. The proposed energetic framework is intended to operate in this manner by introducing an additional systems-level physiological dimension without altering the established computational, informational, or network principles underlying contemporary theories of consciousness. Accordingly, the framework seeks to provide a common physiological context within which multiple existing theories may be further investigated rather than advocating a competing explanation.

Importantly, the present framework should not be interpreted as a competing theory of consciousness. It does not attempt to explain subjective experience, identify a neural correlate of consciousness, or replace existing computational or information-based models. Instead, it proposes that systems-level energetic organization may represent an additional systems-level physiological constraint operating across multiple theoretical frameworks. If supported experimentally, such a perspective could enrich rather than diminish existing models by providing a complementary biological context within which their proposed mechanisms operate.

At present, there is insufficient evidence to determine whether systems-level energetic organization contributes independently to the large-scale neural integration associated with conscious processing or whether its influence is fully accounted for by existing theories (Seth and Bayne, 2022). This uncertainty represents one of the principal motivations for the present hypothesis. Rather than advocating a particular theoretical position, the framework generates experimentally testable predictions capable of distinguishing whether systems-level energetic organization provides explanatory value beyond current models. The following section develops these predictions and considers how they may be experimentally distinguished from competing explanations.

## 5 Testable predictions

A central strength of any scientific hypothesis is its ability to generate predictions that distinguish it from competing explanations and permit empirical evaluation (Popper, 2005). The proposed systems-level energetic framework is intended to satisfy this criterion. The following predictions are derived directly from the proposed systems-level energetic framework and are intended to distinguish the present hypothesis from existing theoretical explanations. These predictions do not constitute supporting evidence for the framework; rather, they identify observations that would be expected if systems-level energetic organization contributes meaningfully to the large-scale neural integration associated with conscious processing.

### 5.1 Prediction 1: systems-level energetic organization will provide explanatory value beyond local metabolic activity

If systems-level energetic organization contributes to conscious processing, measures reflecting the coordinated organization of energetic resources across distributed neural systems should provide explanatory value beyond that offered by measures of isolated regional metabolism alone. This prediction extends beyond demonstrating that active brain regions consume energy. Instead, it proposes that the coordinated organization of energetic processes across interacting neural networks provides additional explanatory information regarding the neural conditions associated with conscious processing.

This prediction differs from existing theories by focusing not on information processing itself, but on the coordinated physiological organization that may support distributed neural integration. It could be evaluated through multimodal experimental approaches combining functional neuroimaging, metabolic imaging, electrophysiology, and computational network analysis (He and Liu, 2008; Calhoun et al., 2014). Statistical models demonstrating independent or incremental predictive value beyond conventional measures of neural activity and regional metabolism would provide support for this prediction (Bullmore and Sporns, 2009; Rubinov and Sporns, 2010).

### 5.2 Prediction 2: disruption of systems-level energetic organization will be associated with reduced large-scale neural integration

The proposed framework predicts that experimental or naturally occurring conditions associated with impaired coordinated systems-level energetic organization will also be associated with measurable reductions in large-scale neural integration. Such changes may occur during general anesthesia, disorders of consciousness, sleep, neurodegenerative disease, or other physiological conditions accompanied by altered brain energetics (Lord et al., 2013; Mashour et al., 2020; Al-Shama et al., 2024). Importantly, this prediction does not suggest that energetic disruption alone determines conscious state. Rather, it predicts that impaired energetic organization will be associated with corresponding alterations in the distributed neural interactions required for conscious processing.

The distinguishing feature of this prediction is its emphasis on coordinated energetic organization rather than isolated changes in regional metabolic activity. Experimental support would therefore require demonstrating relationships between systems-level energetic organization and distributed network integration that cannot be fully explained by local metabolic changes alone (Bullmore and Sporns, 2009; Rubinov and Sporns, 2010).

### 5.3 Prediction 3: preservation of systems-level energetic organization will support stable large-scale neural integration despite regional variability

The framework further predicts that distributed neural integration may remain relatively stable when systems-level energetic organization is preserved, even when regional patterns of neural activity vary substantially. This prediction follows from the hypothesis that coordinated energetic organization operates at the systems level rather than being determined solely by activity within individual brain regions. Accordingly, transient regional fluctuations in neural activity would not necessarily predict disruption of large-scale neural integration provided coordinated systems-level energetic organization remains preserved. Maintaining coordinated energetic support across distributed neural systems may therefore contribute to preserving large-scale neural integration despite local physiological variability.

Testing this prediction would require longitudinal or state-dependent multimodal studies capable of simultaneously evaluating metabolic organization, functional connectivity, electrophysiological dynamics, and network architecture across changing physiological conditions (He and Liu, 2008; Bassett and Sporns, 2017; Preti et al., 2017).

### 5.4 Prediction 4: systems-level energetic measures will provide incremental explanatory value beyond existing network metrics

The proposed framework predicts that quantitative measures describing systems-level energetic organization will provide statistically significant explanatory value beyond established measures of functional connectivity, structural connectivity, oscillatory synchronization, and network topology. If energetic organization represents an independent physiological dimension of large-scale neural integration, incorporating appropriate energetic metrics should improve models of conscious neural processing beyond existing network measures alone.

Support for this prediction would require demonstrating incremental explanatory value after accounting for conventional neurophysiological, metabolic, and network-based variables. Failure to demonstrate such added value would weaken the proposed framework.

### 5.5 Prediction 5: experimental manipulation of systems-level energetic organization will influence large-scale neural integration

A distinguishing prediction of the proposed framework is that experimental interventions capable of modifying systems-level energetic organization, without primarily targeting the information-processing architecture of neural networks, should be associated with measurable changes in large-scale neural integration. This prediction does not assume that energetic organization alone determines conscious processing, nor does it imply that information-processing mechanisms become unimportant. Rather, it predicts that experimentally altering coordinated energetic organization may influence the physiological conditions supporting distributed neural integration in ways that are detectable using multimodal neurophysiological measurements.

Testing this prediction would require carefully designed experimental paradigms capable of independently assessing changes in energetic organization and large-scale neural integration while controlling for established determinants of neural activity. Failure to observe measurable effects under appropriately controlled conditions would weaken support for the proposed framework.

## 6 Falsification criteria

A defining characteristic of a scientific hypothesis is that it can be challenged by empirical evidence. Accordingly, the proposed systems-level energetic framework must generate observations that, if consistently demonstrated, would weaken, require substantial revision of, or falsify its central hypothesis. The following criteria are therefore presented not as expected outcomes, but as conditions under which the proposed framework would lose explanatory value. Explicitly identifying these criteria ensures that the hypothesis remains scientifically testable and avoids becoming unfalsifiable (Popper, 2005).

### 6.1 Criterion 1: no independent relationship between systems-level energetic organization and large-scale neural integration

The central hypothesis would be substantially weakened if rigorous experimental studies consistently demonstrated that measures of systems-level energetic organization provide no statistically significant independent explanatory value beyond established measures of neural activity, functional connectivity, structural connectivity, or network dynamics. If energetic organization contributes no measurable information once conventional neurophysiological variables are considered, the proposed framework would offer little additional explanatory benefit.

Such findings would not invalidate the importance of brain energetics for normal physiology but would argue against the hypothesis that coordinated energetic organization represents an additional systems-level constraint relevant to conscious neural integration.

### 6.2 Criterion 2: large-scale neural integration is unaffected by experimentally verified changes in systems-level energetic organization

The framework predicts that experimentally verified alterations in systems-level energetic organization should be associated with corresponding changes in large-scale neural integration. Consequently, the hypothesis would be weakened if carefully controlled experimental manipulations capable of modifying coordinated energetic organization consistently failed to demonstrate measurable effects on distributed neural integration.

This criterion requires appropriately designed studies that distinguish systems-level energetic organization from generalized metabolic disruption or nonspecific physiological effects. Failure to observe any relationship under such conditions would reduce support for the proposed framework.

### 6.3 Criterion 3: existing theories fully explain observed findings without reference to energetic organization

The proposed framework is intended to complement existing theories rather than replace them. Nevertheless, if all observations attributed to systems-level energetic organization can be fully and parsimoniously explained by established theoretical frameworks without requiring energetic organization as an additional physiological dimension, the rationale for introducing the proposed framework would be substantially diminished.

In this circumstance, the framework would offer limited explanatory value beyond existing models and would require significant revision or abandonment.

### 6.4 Criterion 4: systems-level energetic measures fail to improve predictive models

The hypothesis predicts that incorporating quantitative measures of systems-level energetic organization should improve models of large-scale neural integration beyond established neurophysiological and network-based measures alone. Accordingly, consistent failure across multiple independent datasets to demonstrate statistically significant incremental explanatory or predictive value would weaken the proposed framework.

This criterion emphasizes that the framework must provide measurable scientific benefit rather than simply introducing an additional conceptual perspective.

### 6.5 Criterion 5: proposed relationships cannot be replicated across independent studies

Scientific hypotheses gain credibility through reproducibility. If initial observations supporting relationships between systems-level energetic organization and large-scale neural integration cannot be replicated across independent laboratories, experimental paradigms, or analytical approaches, confidence in the proposed framework would be substantially reduced.

Conversely, reproducible findings across multiple experimental settings would strengthen confidence in the hypothesis without, by themselves, proving its correctness (Munafò et al., 2017).

### 6.6 Criterion 6: energetic influences are demonstrated to be exclusively local rather than systems-level

The central hypothesis specifically proposes that coordinated energetic organization operates at the systems level rather than being fully explained by isolated local metabolic processes. Accordingly, the framework would be substantially weakened if future studies consistently demonstrated that energetic influences on neural function are completely explained by local cellular or regional metabolic processes without evidence of coordinated systems-level energetic organization.

Under such circumstances, energy metabolism would remain fundamental to normal brain physiology, but the principal claim of this manuscript—that systems-level energetic organization represents an additional biological constraint on large-scale neural integration associated with conscious processing—would no longer be supported.

### 6.7 Limitations of falsification

It is important to recognize that failure to support one prediction does not necessarily invalidate every aspect of the proposed framework. Individual predictions may require refinement as scientific understanding of brain energetics and large-scale neural integration evolves. However, consistent failure across multiple independent predictions, particularly those demonstrating independent explanatory value beyond existing theories, would collectively argue against the central hypothesis that systems-level energetic organization represents an additional biological constraint on conscious neural integration.

Accordingly, the proposed framework should be regarded as provisional and subject to continual refinement or rejection based upon empirical evidence. This commitment to explicit falsifiability reflects the principles of a Hypothesis & Theory article and provides the foundation for the experimental directions described in the following section.

## 7 Future experimental directions

Having established explicit falsification criteria, the next step is to define experimental strategies capable of evaluating the proposed systems-level energetic framework. The studies described below are intended not to confirm the hypothesis, but to determine whether it withstands rigorous empirical testing. Throughout these investigations, three levels of inference should remain clearly distinguished: (1) established physiological effects, (2) plausible mechanistic relationships between physiology and systems-level energetic organization, and (3) the hypothesis that coordinated energetic organization contributes to the physiological conditions supporting distributed neural integration. Collectively, these studies should determine whether measures of systems-level energetic organization provide reproducible explanatory or predictive value beyond established neurophysiological, neuroimaging, and computational measures. Consistent improvement across independent cohorts, laboratories, and experimental paradigms would justify continued investigation, whereas persistent failure to demonstrate incremental explanatory value would weaken the proposed framework.

### 7.1 Experimental principles

Experimental evaluation should employ multimodal study designs capable of assessing systems-level energetic organization alongside neural activity, functional and structural connectivity, oscillatory dynamics, regional metabolism, cerebral perfusion, autonomic physiology, behavior, and large-scale network organization. Multimodal approaches are particularly valuable because electrophysiological, hemodynamic, metabolic, autonomic, and behavioral techniques each provide complementary temporal, spatial, and physiological information (He and Liu, 2008).

Energetic organization should be operationalized using prespecified quantitative variables rather than inferred solely from regional metabolic activity or isolated neuronal activation. Throughout this framework, energy and energetics refer to physiological and metabolic energetic states and processes and are distinct from variational free energy as used in predictive-processing and active-inference formulations. Candidate energetic state variables may include measures of mitochondrial redox state, ATP-related dynamics, glucose metabolism, and oxygen metabolism, selected according to the spatial and temporal scale of the experiment. In appropriate experimental preparations, established optical approaches such as NAD(P)H/FAD redox imaging and fluorescence-lifetime imaging (FLIM), together with validated genetically encoded ATP reporters where applicable, may provide spatially and temporally resolved indices of cellular energetic state (Kolenc and Quinn, 2019; White and Yang, 2022). These measurements should not be interpreted individually as systems-level energetic organization. Rather, the systems-level construct should be quantified from the spatial patterning, temporal coordination, variability, stability, and coupling of energetic states across distributed neural populations. Complementary measures of cerebral perfusion, neurovascular or neurometabolic coupling, and neural activity can then be acquired simultaneously to determine how energetic organization relates to the physiological support and neural dynamics of large-scale integration (Lord et al., 2013; Klug et al., 2024; Rae et al., 2024).

Large-scale neural integration should likewise be operationalized using prespecified network-level measures rather than inferred from neural activity alone. In this framework, large-scale neural integration refers to coordinated interactions among spatially distributed and functionally differentiated neural populations. Candidate measures may include interregional functional connectivity, network integration and segregation, between-module connectivity, hub or centrality measures, and dynamic changes in network configuration over time. The specific metrics should be selected according to the experimental modality and hypothesis rather than treated as interchangeable measures of integration. No individual network metric is assumed to constitute large-scale neural integration; where feasible, convergent measures should be used to determine whether distributed neural populations exhibit coordinated interactions across differentiated neural systems (Bullmore and Sporns, 2009; Rubinov and Sporns, 2010; Bassett and Sporns, 2017; Preti et al., 2017).

Because neural activity and brain energetics are physiologically coupled, the proposed framework does not require their complete biological decoupling. Instead, experimental designs should seek partial dissociations where feasible while simultaneously acquiring energetic and neural measurements (Trevisiol et al., 2017). Analyses should compare models based on established neural variables with otherwise comparable models that additionally include prespecified measures of energetic organization, thereby testing whether energetic variables provide reproducible incremental explanatory or predictive value beyond neural activity, functional and structural connectivity, oscillatory dynamics, regional metabolism, and network topology. Where experimentally feasible, perturbations of defined energetic processes may provide a stronger test by determining whether altered energetic organization is associated with predicted changes in large-scale neural integration while neural activity and relevant physiological covariates are measured concurrently (Elzoheiry et al., 2020). Temporal analyses should additionally determine whether changes in energetic organization precede, accompany, or follow changes in neural integration. Dynamic-connectivity analysis and network-science methodologies may be particularly useful for evaluating time-varying systems-level organization across changing brain states (Bassett and Sporns, 2017; Preti et al., 2017).

A particularly stringent causal test would attempt to alter the spatial or temporal organization of energetic states across distributed neural populations while matching overall energetic availability and regional mean energetic measures as closely as experimentally feasible. Energetic and neural measurements should be acquired simultaneously to verify that the perturbation altered energetic organization rather than merely total or regional energetic state and to quantify the corresponding effects on large-scale neural integration. The framework predicts that verified disruption of energetic organization should produce a prespecified change in large-scale neural integration even when gross energetic availability is relatively preserved. Conversely, if large-scale neural integration remains intact despite reproducible disruption of energetic organization under appropriately matched conditions, this would provide strong evidence against the proposed systems-level energetic constraint.

Primary hypotheses, outcome measures, statistical analyses, and falsification thresholds should be prospectively specified whenever feasible. Study preregistration, transparent reporting, data sharing where appropriate, independent replication, and validation across multiple cohorts and laboratories should be emphasized over isolated statistically significant findings (Munafò et al., 2017).

### 7.2 Human experimental studies

Human investigations provide the most direct opportunity to determine whether measures of systems-level energetic organization independently contribute to explaining distributed neural integration under physiologically and clinically relevant conditions.

Appropriate multimodal assessments may include electroencephalography, magnetoencephalography, functional and diffusion magnetic resonance imaging, arterial spin-labeling perfusion imaging, positron-emission tomography, magnetic resonance spectroscopy, autonomic physiology, behavioral performance, and computational network analysis. Simultaneous acquisition of complementary modalities is preferable when technically feasible, although carefully standardized longitudinal acquisition may be appropriate when simultaneous measurement is impractical.

Suitable paradigms include transitions among wakefulness, sleep, general anesthesia, pharmacologically altered states, disorders of consciousness, cognitive-task performance, healthy aging, and neurological recovery (Mashour et al., 2020). These conditions are appropriate because they permit controlled or repeated assessment of neural integration and energetic physiology, not because any individual condition is presumed to validate the proposed framework.

When experimental hypotheses concern conscious state or conscious processing, these variables should be operationalized independently of the energetic and neural-integration measures under investigation. Depending on the paradigm, this may involve prespecified contrasts between conscious and nonconscious states or between consciously and nonconsciously processed contents, together with appropriate behavioral or subjective measures where available. Energetic organization or large-scale neural integration should not themselves be used to define the conscious condition being tested (Mashour et al., 2020).

Primary analyses should determine whether candidate energetic measures explain variance in large-scale neural integration after accounting for established measures of neural activity, network structure, connectivity, oscillatory dynamics, and regional metabolism. Temporal analyses should additionally evaluate whether changes in energetic organization precede, accompany, or follow changes in distributed neural integration.

Studies should include appropriate control conditions while accounting for potential confounding factors including age, medication exposure, vascular disease, sleep quality, physical activity, cardiorespiratory fitness, metabolic status, and neurological diagnosis. Whenever possible, prospective preregistration and within-participant repeated-measures designs should be incorporated to improve reproducibility and reduce interindividual variability.

### 7.3 Non-human animal experimental studies

When non-human animal models are used, they may permit physiological and circuit-level manipulations not currently achievable in humans; such approaches should be adopted only where non-invasive alternatives are insufficient to test the causal predictions at hand, and should be conducted under rigorous ethical oversight and welfare safeguards.

Experiments may combine electrophysiology, cerebral blood-flow measurement, mitochondrial assessment, metabolic imaging, molecular analyses, autonomic physiology, and behavioral assessment across multiple neural regions. Optogenetic, chemogenetic, pharmacological, genetic, or physiological approaches may be incorporated when they permit selective manipulation of defined circuits or energetic processes, provided that biological specificity and potential off-target effects are carefully characterized (Mirabella and Fenselau, 2023).

These studies should explicitly distinguish localized metabolic responses from coordinated systems-level energetic organization. Strong experimental designs would manipulate a defined physiological component, quantify its effects across multiple regions and modalities, and determine whether resulting changes in energetic organization predict alterations in distributed neural integration.

The physiological relationship between neural activity and brain energetics is inherently bidirectional: neural activity alters metabolic demand and energetic state, while the present hypothesis asks whether the resulting organization of energetic states may in turn constrain distributed neural integration. Temporal ordering therefore remains an empirical question. Demonstrating that coordinated energetic changes consistently precede alterations in distributed neural integration would strengthen the proposed constraint relationship. Simultaneous changes would demonstrate association but not directionality, whereas neural integration changes consistently preceding energetic reorganization would challenge a unidirectional energetic-to-neural interpretation and require refinement of the proposed mechanistic relationship. Cross-species investigations should additionally determine whether candidate energetic metrics remain meaningful across differences in brain size, architecture, metabolism, behavioral repertoire, and state regulation. Convergent findings across species would strengthen the framework, whereas highly species-specific relationships lacking biological explanation would weaken its generalizability.

### 7.4 Computational modeling

Computational neuroscience provides an additional means of evaluating the proposed framework without replacing established neural mechanisms. Computational tests need not require comprehensive whole-brain simulation or simultaneous representation of all cellular, metabolic, and network processes. Instead, they may employ multiscale abstractions selected according to the hypothesis, available data, and spatial and temporal scale of interest. Depending on the modeling scale, existing network or whole-brain models may be extended by incorporating variables describing energetic production, allocation, distribution, utilization, regulation, and energetic costs associated with transitions among network states (Lang et al., 2024).

The relevant scientific question is not whether neural computation requires energy, which is already well established, but whether explicit energetic variables improve prediction of large-scale neural dynamics beyond models based on structural connectivity, functional connectivity, regional activation, oscillatory activity, and network topology (Gu et al., 2018).

A minimal quantitative formulation can represent systems-level energetic organization as E(t), defined by prespecified spatial and temporal properties of measured energetic states across distributed neural populations, and large-scale neural integration as N(t), quantified using prespecified network measures. The central hypothesis can then be evaluated by comparing a baseline model, N(t) = f[X(t)], with an energetically extended model, N(t) = f[X(t), E(t)], where X(t) represents established neural variables and relevant non-energetic covariates. The framework does not prescribe a specific functional form for f or assume that E(t) is independent of X(t). Rather, it predicts that inclusion of E(t) should provide reproducible explanatory or predictive information beyond the baseline variables if systems-level energetic organization constitutes an additional constraint on large-scale neural integration.

Computational implementations may also examine energetic organization within models containing hierarchical or latent-state dynamics. Predictive-coding-inspired models provide examples in which prediction errors, hierarchical interactions, and latent internal states can contribute to transitions among modes of processing (Oyama et al., 2025), while active-inference approaches provide a broader computational framework for relating hierarchical inference to conscious processing (Laukkonen et al., 2025). Within the present framework, energetic variables could be incorporated as additional state variables or constraints within such models to test whether their inclusion improves prediction of changes in large-scale neural integration beyond neural or computational variables alone. This approach does not assume that energetic organization constitutes the computational content of prediction errors or latent states; rather, it provides a means of testing whether physiological energetic organization constrains the neural implementation and dynamics of such computational processes.

Computational evaluation should compare nested models, incorporate out-of-sample prediction, penalize unnecessary model complexity, and assess robustness across parameter selections and independent datasets. Model comparison should favor parsimony, retaining energetic variables only when they produce reproducible explanatory or predictive improvement beyond established neural variables (Durstewitz et al., 2023).

Models should also distinguish energetic cost from energetic organization. A system may consume substantial energy without exhibiting coordinated energetic distribution, whereas a comparatively efficient system may maintain stable integration through tightly regulated allocation across regions and timescales. This distinction should be explicitly represented within computational model design.

Failure of energetic variables to produce reproducible predictive improvement would argue against their inclusion as an independent systems-level constraint. Conversely, consistent improvement across models, datasets, and physiological states would justify continued empirical investigation without establishing that energetic organization alone explains conscious processing.

### 7.5 Translational physiological studies

Translational physiological interventions may serve as experimental probes of the proposed framework, but they should not be interpreted as evidence that the framework is correct. Instead, they provide opportunities to investigate whether established physiological changes are accompanied by measurable alterations in candidate indices of systems-level energetic organization and whether those changes independently relate to distributed neural integration.

Central physiological probes include cardiovascular exercise and sleep optimization. Cardiovascular exercise influences cardiorespiratory fitness, vascular function, cerebral perfusion, autonomic regulation, metabolic health, inflammatory signaling, and neuroplasticity, while sleep–wake regulation influences neural communication, cerebral metabolism, autonomic physiology, and large-scale brain dynamics (Al-Shama et al., 2024; Walzik et al., 2024). These established physiological effects make both interventions suitable for investigating whether coordinated energetic organization provides explanatory value beyond conventional physiological and neurophysiological measures.

Cellular and metabolic probes include photobiomodulation and pulsed electromagnetic field therapy (PEMF). Photobiomodulation has been associated with mitochondrial signaling, cellular redox regulation, adenosine triphosphate production, reactive oxygen species, and nitric oxide-related pathways under defined exposure conditions (Maghfour et al., 2024). PEMF may likewise influence cellular physiology, although evidence regarding its effects on systems-level energetic organization remains comparatively limited. Neither intervention should be interpreted as directly altering consciousness; rather, each provides experimentally testable physiological targets requiring rigorous sham-controlled investigation (Ryu et al., 2024).

Peripheral mechanobiological and neurophysiological probes include physiotherapy, acupuncture, extracorporeal shockwave therapy (ESWT), therapeutic ultrasound, and related evidence-supported interventions. These therapies may influence mechanotransduction, vascular signaling, nociceptive processing, autonomic regulation, tissue repair, and peripheral neurophysiology (Demuynck et al., 2024). Their established physiological effects do not demonstrate changes in systems-level energetic organization or conscious processing, but they provide biologically plausible opportunities to investigate whether peripheral physiological modulation is associated with coordinated central energetic changes when evaluated using multimodal experimental designs.

The proposed framework does not assert that exercise, physiotherapy, photobiomodulation, acupuncture, ESWT, therapeutic ultrasound, PEMF, or sleep optimization directly alter consciousness or validate the hypothesis. Rather, it predicts that interventions producing measurable physiological adaptations may serve as experimental probes for determining whether coordinated systems-level energetic organization also changes and whether those changes independently relate to distributed neural integration.

Future translational studies should therefore employ intervention-specific protocols, rigorous sham or control conditions whenever feasible, prespecified physiological targets, prospective statistical analyses, and multimodal outcome measures. Positive findings would require more than symptomatic or functional improvement; candidate energetic variables would need to demonstrate reproducible explanatory value beyond established physiological and neurophysiological measures.

### 7.6 Clinical rehabilitation studies

Clinical rehabilitation provides a valuable translational research environment because it permits repeated physiological assessment during progressive functional adaptation while delivering interventions at clinically relevant doses. This setting is particularly well suited for longitudinal multimodal investigations of the proposed framework (Cardoso et al., 2024).

A feasible human study could evaluate participants undergoing standardized rehabilitation programs incorporating cardiovascular exercise, physiotherapy, and selected evidence-supported therapeutic modalities. Repeated assessments could combine electroencephalography or magnetoencephalography, functional or perfusion imaging, metabolic imaging where appropriate, autonomic physiology, computational network analysis, validated behavioral measures, and functional outcome assessments. For clarity, outcome measures should be interpreted according to their level of inference.

Direct hypothesis-related measures include electroencephalography, magnetoencephalography, functional magnetic resonance imaging, arterial spin-labeling, positron-emission tomography, magnetic resonance spectroscopy, and computational measures of large-scale neural integration. These techniques provide the most direct evaluation of the manuscript’s central hypothesis.

Indirect translational measures include infrared thermography, cardiorespiratory physiology, muscle oxygenation, tissue-perfusion assessment, gait analysis, balance testing, functional mobility, autonomic measurements, and validated clinical outcome instruments. These variables do not directly measure consciousness or large-scale neural integration but may characterize systemic physiological adaptation when interpreted alongside central neurophysiological measurements.

The primary endpoint should not be whether rehabilitation alters consciousness. Rather, studies should determine whether rehabilitation produces coordinated changes in prespecified energetic measures and whether those changes independently predict alterations in distributed neural integration after accounting for established physiological and neurophysiological variables. Clinical improvement alone would not constitute evidence supporting the proposed framework.

A staged research strategy is recommended. Initial feasibility studies should establish recruitment, adherence, tolerability, protocol fidelity, measurement reliability, and preliminary effect-size estimates. Subsequent controlled investigations should evaluate temporal mediation, dose–response relationships, incremental predictive value, durability of physiological change, and reproducibility across independent research sites.

Whenever possible, studies should distinguish the physiological effects of individual interventions from those of multimodal rehabilitation programs. Factorial, sequential, adaptive, or carefully controlled pragmatic study designs may help determine whether observed physiological changes result from specific interventions, cumulative treatment exposure, increased physical activity, therapeutic interaction, or broader behavioral adaptation.

Collectively, these experimental strategies position the proposed framework as a testable scientific research program rather than a conceptual assertion. Ultimately, the framework should be judged not by its conceptual appeal, but by whether rigorous, reproducible, multimodal investigations demonstrate that systems-level energetic organization provides independent explanatory value beyond established models of large-scale neural integration. Failure to meet this standard would require refinement or rejection of the framework, whereas consistent support across independent investigations would justify its continued scientific development.

## 8 Conclusion

Conscious processing is associated with coordinated interactions among distributed neural systems, and these interactions necessarily operate within the energetic constraints of brain physiology (Rae et al., 2024). Contemporary theories have substantially advanced understanding of the computational, informational, and network mechanisms associated with conscious processing (Mashour et al., 2020; Seth and Bayne, 2022). However, whether the coordinated organization of energetic resources across distributed neural systems represents an additional biological constraint on large-scale neural integration remains incompletely understood.

This manuscript proposes a systems-level energetic framework to address this unresolved question. The central hypothesis is that the coordinated organization of energetic resources across distributed neural systems is hypothesized to constrain the capacity for large-scale neural integration associated with conscious processing. The framework does not propose that energy generates consciousness, determines conscious content, or explains subjective experience. Rather, it considers energetic organization as a complementary physiological dimension operating alongside the mechanisms emphasized by existing theories.

The framework is grounded in established principles of brain energetics, metabolic regulation, and distributed neural organization, while the proposed relationship between their systems-level energetic organization and conscious neural integration remains hypothetical. Its novelty lies not in proposing new metabolic or neural mechanisms, but in asking whether the coordinated organization of established energetic processes across distributed neural systems provides independent explanatory value for understanding large-scale neural integration (Bullmore and Sporns, 2009; Harris et al., 2012).

The hypothesis generates experimentally distinguishable predictions and explicit conditions under which it should be weakened, refined, or rejected. In particular, failure of systems-level energetic measures to provide reproducible explanatory or predictive value beyond established neurophysiological and network variables would argue against the framework. Testing this proposition will require rigorous multimodal investigation across human and non-human animal studies, computational modeling, and carefully controlled translational paradigms. Physiological and rehabilitation interventions may serve as indirect experimental probes, but their effects should not be interpreted as evidence that they directly alter consciousness or validate the hypothesis.

Ultimately, the scientific value of the proposed framework will depend on empirical performance rather than conceptual appeal. If systems-level energetic organization demonstrates reproducible explanatory or predictive value beyond established models across independent experimental paradigms, it may provide a useful complementary dimension for understanding the biological constraints supporting conscious neural processing. If it does not, the framework should be refined or rejected. By framing systems-level energetic organization in explicitly testable and falsifiable terms, the proposed framework provides a research program for investigating an unresolved dimension of large-scale neural integration while remaining agnostic regarding the ultimate origin and nature of subjective experience.

## Data Availability

The original contributions presented in the study are included in the article/supplementary material, further inquiries can be directed to the corresponding author.
